# Cortical Thinning in Network-Associated Regions in Cognitively Normal and Below-Normal Range Schizophrenia

**DOI:** 10.1155/2017/9760905

**Published:** 2017-02-28

**Authors:** R. Walter Heinrichs, Farena Pinnock, Melissa Parlar, Colin Hawco, Lindsay Hanford, Geoffrey B. Hall

**Affiliations:** ^1^York University, Toronto, ON, Canada; ^2^Centre for Addiction and Mental Health, Toronto, ON, Canada; ^3^University of Pittsburgh, Pittsburgh, PA, USA; ^4^McMaster University, Hamilton, ON, Canada

## Abstract

This study assessed whether cortical thickness across the brain and regionally in terms of the default mode, salience, and central executive networks differentiates schizophrenia patients and healthy controls with normal range or below-normal range cognitive performance. Cognitive normality was defined using the MATRICS Consensus Cognitive Battery (MCCB) composite score (*T* = 50 ± 10) and structural magnetic resonance imaging was used to generate cortical thickness data. Whole brain analysis revealed that cognitively normal range controls (*n* = 39) had greater cortical thickness than both cognitively normal (*n* = 17) and below-normal range (*n* = 49) patients. Cognitively normal controls also demonstrated greater thickness than patients in regions associated with the default mode and salience, but not central executive networks. No differences on any thickness measure were found between cognitively normal range and below-normal range controls (*n* = 24) or between cognitively normal and below-normal range patients. In addition, structural covariance between network regions was high and similar across subgroups. Positive and negative symptom severity did not correlate with thickness values. Cortical thinning across the brain and regionally in relation to the default and salience networks may index shared aspects of the psychotic psychopathology that defines schizophrenia with no relation to cognitive impairment.

## 1. Introduction

Cognitive impairment is highly prevalent in schizophrenia, with dysfunction across multiple abilities observed in 75–80% of patients [1]. Nonetheless, a minority overlaps with the performance of healthy control participants, giving rise to the possibility of an illness variant free, or relatively free, of cognitive deficits. It is likely that cognitive performance forms a continuum in the patient population, ranging from impaired to normative values, rather than a discrete or binary disease marker. However, this does not obviate the potential benefit of studying patients with psychosis who are relatively free of cognitive impairment. These exceptional patients may represent important variations in underlying pathophysiology and disease compensation. At the same time, the validity of “true” cognitive normality in schizophrenia has been disputed based on conjectures that normal range performance in patients represents a decline from premorbid ability levels [2]. In addition, putatively normal range patients may demonstrate task deficits and discrepant performance profiles when compared directly with healthy control groups [3, 4]. This is not always the case [5] and absolute performance normality in any clinical population that endures a substantial stress and illness burden may be an unsupportable expectation.

Cognitive impairment and severe psychopathology both implicate underlying disturbances in neural systems. Substantial effort has been devoted to finding the biological underpinnings of schizophrenia through application of neuroimaging techniques. Structural neuroimaging studies have reported widespread reductions in grey matter volume and cortical thickness in the illness [6–9]. Cortical thinning is heritable and associated with specific genes and pathways that may confer risk for psychosis [10]. However, it is unclear whether these structural reductions index psychotic psychopathology, cognitive impairment, or both. Behavioral data support the possibility that psychosis and cognition are distinct and dissociable, but neuroimaging data are more equivocal. Structural imaging findings have been related to both symptoms and cognitive performance and grey matter reductions in specific regions have shown substantial variability [9]. Neurobiological evidence bearing on the validity of a cognitively normal or near-normal disease variant is scant and inconsistent. Grey matter volumes are lower in both cognitively normal and below-normal range patients relative to controls, implying that cortical changes are a central illness feature tied to the defining psychopathology of schizophrenia [11]. In contrast, recent data indicate that cortical thinning occurs primarily in cognitively impaired patients and minimally in patients with normal or near-normal cognitive performance [12, 13]. Against this, another report showed no differences in grey matter in patients relative to controls, but both cognitively normal and below-normal range patients demonstrated reduced white matter volumes [14].

In light of these considerations, we asked whether cortical thinning (1) is primarily a shared feature of patients with schizophrenia and therefore primarily an index of psychotic psychopathology or (2) reflects the presence of cognitive impairment in the large subgroup of patients with deficient performance and/or (3) reflects an interaction among disease processes. To answer these questions, we evaluated cortical thickness in schizophrenia patients as well as in healthy control participants meeting and failing to meet a criterion for cognitive normality based on a widely used neuropsychological test battery [15]. It is noteworthy that the low-performing region of the general population distribution is seldom accessed to establish control comparisons in schizophrenia research [16]. Accordingly, little is known about structural brain differences between patients and cognitively low-performing but psychiatrically unremarkable participants. This kind of comparison may reveal neural characteristics intrinsic to schizophrenia and eliminate those that occur as a function of general ability level across populations.

In addition to comparing cortical thickness values across the whole brain, we focused on regions associated with the default mode network (DMN), central executive network (CEN), and salience network (SN). Each of these networks and their interrelations have been implicated in severe forms of psychopathology including schizophrenia [17]. The DMN comprises primarily ventromedial prefrontal and posterior cingulate cortex and mediates self-referential thinking, including aspects of autobiographical memory and social cognition [18]. The CEN comprises regions of dorsolateral prefrontal and posterior parietal cortex and is involved with regulating attention during cognitive task performance [19]. The SN includes ventrolateral prefrontal and anterior insula and dorsal anterior cingulate cortical regions and contributes to the detection of stimulus significance and may also play a coordinating role in terms of the other two networks [20]. An overactive DMN coupled with aberrant salience mapping and reduced CEN activity during information processing has been posited as an underlying defect in disorders that involve severe psychopathology and cognitive impairment [17]. Reduced surface area has been reported for cortical regions associated with these networks in schizophrenia patients, but it is not known whether this is true across the cognitive impairment/normality distinction [21]. Accordingly, our data address the additional question of the extent to which cortical thickness values for key brain systems are shared or different across schizophrenia patients and healthy controls with normal range and below-normal range cognitive performance.

## 2. Materials and Methods

### 2.1. Participants

Patients (*n* = 90) were recruited from several outpatient programs in Hamilton, Ontario, Canada: the Cleghorn Early Intervention Clinic (St. Joseph's Healthcare Hamilton), the Hamilton Program for Schizophrenia, the Schizophrenia Outpatient Clinic (St. Joseph's Healthcare Hamilton), Schizophrenia Services of Ontario, Hamilton Chapter, Path Employment Services, and the Wellington Psychiatric Outreach Program. Criteria for study entry included (1) a diagnosis of schizophrenia or schizoaffective disorder confirmed by the Structured Clinical Interview for DSM-IV Axis I Disorders [22], with no concurrent diagnosis of substance use disorder; (2) a history free of developmental or learning disability; (3) a history free of neurological or endocrine disorder; and (4) age 18–65. Healthy control participants (*n* = 63) were recruited through local newspaper and online classified advertisements for paid research participation. To maximize the probability of recruiting control participants with below average range cognitive functioning, advertisements were targeted to community, employment, and social service agencies oriented to unskilled and less educated populations. Interested individuals were screened for psychiatric history and substance use disorders. All participants provided written informed consent and the research was approved by institutional ethics review boards.

### 2.2. Cognitive Measures and Group Assignment

Standard cognitive tests forming the criterion for performance normality comprised the MATRICS (Measurement and Treatment Research to Improve Cognition in Schizophrenia) Consensus Cognitive Battery (MCCB) [15]. The MCCB includes individual measures of working memory, attention, verbal memory, processing speed, reasoning and problem-solving, visual learning, and social cognition and yields a composite index of overall performance. In addition, the Reading subtest of the Wide Range Achievement Test (WRAT-4) was administered as a proxy measure of premorbid ability [23]. Clinical status of patient participants was assessed with the Positive and Negative Syndrome Scale (PANSS) [24].

Group assignment was based on MCCB composite scores summarizing performance across 7 ability domains, with a *T* score of 50 ± 10 representing normative mean performance in the community standardization sample and in line with previous studies using this instrument [5]. Accordingly, the criterion for assignment to cognitively normal range groups was an overall composite *T* score from 40 to 60. Participants with a composite *T* score < 40 were assigned to below-normal range groups. Application of this performance criterion to the pool of 90 patients yielded *n* = 17 cognitively normal and *n* = 73 below-normal range patients. However, 24 in the below-normal range group transitioned to inpatient status during the 3-year course of the study and/or were unable or unwilling to complete the MRI imaging protocol, yielding a final *n* = 49 below-normal range patients. The patients who dropped out did not differ significantly from the final group of below-normal range patients in terms of age, symptom severity, or medication. However, the proportion of males in the excluded group (87%) differed significantly (*χ*_1_^2^ = 4.62; *p* = 0.03) from the proportion of males in the study group (63%). The same normality criterion was applied to the pool of healthy controls to yield *n* = 39 cognitively normal and *n* = 24 below-normal range controls.

### 2.3. MRI Imaging

#### 2.3.1. Scan Acquisition

Participants underwent scanning with a 3.0-Tesla whole body short bore General Electric System MRI scanner with an 8-channel parallel receiver head coil at the Imaging Research Centre, St. Joseph's Healthcare Hamilton. A T1-weighted axial anatomical scan was acquired using a three-dimensional fast spoiled gradient recalled echo sequence with inversion recovery preparation. The anatomical image had 152 slices (2 mm thick with 1 mm overlap) with the following imaging parameters: time to repetition (TR)/echo time (TE) = 7.5/2.1 ms, TI = 450 ms, field of view (FOV) = 24 cm, matrix = 512 × 512, flip angle = 12°, receiver bandwidth (rBW) = +/−62.5 kHz, and number of excitations (NEX) = 1.

#### 2.3.2. Cortical Thickness Analysis

The T1-weighted images collected for each participant were preprocessed in order to segment the brain and to align cortical structures across the subjects using FreeSurfer automated image analysis (version 5.1.0; http://surfer.nmr.mgh.harvard.edu/; see [25, 26] for further details on this technique). Each image was inspected to correct for motion and also underwent spatial and intensity normalization and skull stripping. Cortical thickness was defined as the distance between pial surface to the grey/white matter border across 160,000 vertices in both cerebral hemispheres. Subsequently, each image was visually inspected by trained inspectors blind to group assignment to correct inaccuracies. Once images passed inspection, high dimensional registration was used to map them onto a spherical atlas for increased intersubject alignment accuracy. Surface maps were smoothed with a 15 mm full-width-half-maximum Gaussian kernel.

**Figure 1:**
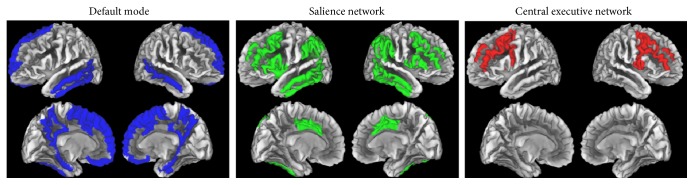
Cortical regions [27] associated with each brain network. Regional overlap between the default mode network (DMN) and salience network (SN) included the left inferior temporal gyrus and middle-anterior cingulate gyrus and sulcus bilaterally. Overlap between the SN network and central executive network (CEN) included the opercular part of the inferior frontal gyrus bilaterally as well as the middle frontal gyrus and left anterior segment of the circular sulcus of the insula. There was no overlap between the DMN and the CEN.

Cortical parcellations were obtained for regions of interest (ROIs) using the methods described by Destrieux et al. [27, 28] in FreeSurfer. The Destrieux atlas involves both gyral and sulcal structures for bilateral hemispheric parcellation. A priori ROIs were chosen for analysis based on three networks (DMN, SN, and CEN; [29]). A visual representation of ROIs associated with each network is available in Figure 1. Traditionally, research has treated these networks as disjoint clusters and imposed assumptions regarding orthogonality. However, recent theory and data show that structural and functional overlap among network regions is more accurate and provides a promising framework for investigation [30]. Accordingly, we included ROIs that were common to more than one network (see Figure 1). In addition, because cortical thickness changes in schizophrenia are widespread or multifocal rather than highly localized, thickness data for ROIs assigned to each network were summed and averaged to yield DMN, SN, and CEN values. This avoided the multiple-comparison problems inherent in whole brain neuroimaging studies [31–33]. Additionally, this ROI-based approach was considered more appropriate than vertex-wise analyses given the heterogeneity and likelihood of widespread but relatively small changes in thickness values typically observed in schizophrenia patients [34, 35].

Box's test for covariance matrix inequality and Levene's tests for variance inequality were performed prior to any parametric statistical testing. A multivariate analysis of covariance (MANCOVA) was carried out on the cortical thickness data, with age as a covariate and cognitive status/diagnosis as the fixed factor.

## 3. Results

Descriptive statistics for the study groups are presented in Table 1. Below-normal range patients were older and less educated than cognitively normal range patients (*t*(64) = 3.50, *p* < 0.01; *t*(64) = 3.81, *p* < 0.01) and controls (*t*(86) = 2.72, *p* < 0.01; *t*(86) = 2.72, *p* < 0.01). In addition, cognitively normal range controls were more educated than below-normal range controls (*t*(61) = 5.77, *p* < 0.001), but less educated than normal range patients (*t*(54) = −2.13, *p* < 0.001). There were no differences in the proportion of males in each group. In terms of MCCB composite scores, as expected, the cognitively normal range patient and control groups did not differ and the below-normal range patient and control groups did not differ. However, cognitively normal range patients differed from below-normal range patients (*t*(64) = 9.61, *p* < 0.001) and controls (*t*(39) = 7.50, *p* < 0.001) and cognitively normal range controls differed from below-normal range patients (*t*(86) = 14.97, *p* < 0.001) and controls (*t*(61) = 11.20, *p* < 0.001). The same pattern was observed in terms of Reading ability (WRAT-4), a proxy or estimate of premorbid ability. The key comparison of cognitively normal range patients with controls revealed no significant difference (*t*(54) = −0.273, *p* = 0.79). Additional details on the cognitive characteristics of the cognitively normal range patients have been published separately [3]. Patient subgroups did not differ in the severity of positive and negative symptoms or in the frequency of second-generation antipsychotic medication.

The MANCOVA on cortical thickness revealed a significant main effect of group (*F*_12,320_ = 2.85, *p* = 0.001, partial *η*2 = 0.085) and a covariate effect for age (*F*_4,121_ = 12.18, *p* < 0.001, partial *η*2 = 0.29). Univariate *F* ratios were significant for whole brain as well as for SN- and DMN-associated regional cortical thickness. Cognitively normal range controls demonstrated significantly higher thickness values than both patient subgroups after Bonferroni adjustment (see Table 2). Partial correlations controlling for age were calculated to index relationships between network thickness values separately for each participant group. This revealed consistently high and significant (*p* < 0.001) correlations for all groups (CNR patients: mean *r* = 0.87, range: *r* = 0.82–*r* = 0.96; CNR controls: mean *r* = 0.83, range: *r* = 0.68–*r* = 0.96; BNR patients: mean *r* = 0.78, range: *r* = 0.69–*r* = 0.84; BNR controls: mean *r* = 0.86, range: *r* = 0.82–*r* = 0.93). Given group differences in educational achievement, this variable was also considered as a potential covariate. However, all bivariate correlations between education and cortical thickness were nonsignificant for both patients and control participants. Moreover, inclusion of education as a covariate in the MANCOVA did not alter the pattern of results described above. There were no significant bivariate correlations between PANSS ratings and regional/network-related or whole brain cortical thickness values.

## 4. Discussion

Our data suggest that cortical thinning across the whole brain, as well as in default mode and salience network-associated regions, is a characteristic of the pathophysiology of schizophrenia and not related to the impaired cognition that also occurs frequently, but not invariably, in the disorder. A small but significant portion of the schizophrenia population meets psychometric criteria for normal range cognitive performance without evidence of decline from preillness levels. This subgroup is thereby distinguished from the large majority of more typical, cognitively impaired, and, frequently, deteriorated patients. Moreover, the cognitive distinction implies corresponding neural differences in cerebral structure and function. However, both patient subgroups demonstrated thinning relative to healthy control participants implying that this aspect of brain structure reflects the primary psychosis-related pathology of schizophrenia. In addition, controls with normal range or below-normal range ability were mutually indistinguishable in terms of cortical thickness. This underscores the relative independence of cortical thickness and cognitive performance. At the same time, overall structural covariance between network-associated regions was consistently high and occurred across cognitive and psychiatric status.

Cognitively normal range patients and controls also differed, perhaps surprisingly, in educational achievement, with patients obtaining on average an extra year relative to control participants. It is known that achievement is significantly lower in cognitively unselected schizophrenia patients than in the general population and relative to other psychiatric populations [36]. However, patients with normal range cognition are relatively rare and represent a special subgroup of individuals. In these cases, education may provide a protective influence in terms of the more typical deficits experienced by schizophrenia patients. Alternatively, cognitively high-functioning patients may be spurred to persist with education as a normalizing coping response as psychotic illness begins and intensifies. Accordingly, it is difficult to specify whether educational achievement is a producer or a product of cognitive proficiency in this population [37].

Our findings are consistent with previous reports that structural aspects of the cerebral cortex differentiate schizophrenia patients from healthy controls regardless of cognitive ability levels [11]. The data thereby contradict evidence that diffuse cortical thinning occurs preferentially or more severely in cognitively impaired patients [13]. Part of the reason for this inconsistency may lie in the nature of the normality criteria used by different researchers and the application of these criteria to patient and control participants. Thus, Cobia and colleagues [13] used cluster analysis to identify a subgroup of patients with “near-normal” performance defined by norm-referenced data values. However, these patients were impaired relative to comparison participants on several tasks. In contrast, Wexler and colleagues [11] used direct comparison with controls as the criterion whereby patients had to perform within 0.5 standard deviations of control values to be defined as “near normal.” More recently, Woodward and Heckers [14] reported no differences in grey matter volumes between cognitively normal range schizophrenia patients, controls, and impaired patients using a psychometric normality algorithm that incorporated estimated premorbid as well as current ability. It seems likely that heterogeneity in normality criteria and definitions contributes to the variability of findings. The use of widely accepted and comprehensive but time-efficient measures like the MCCB may yield more consistent data.

It is noteworthy that grey matter reductions have been demonstrated in prefrontal and medial temporal systems in schizophrenia [38]. However, to our knowledge, the present results are the first to show thinning in cortical areas associated specifically with the default mode and salience networks. Aberrant connectivity and activation patterns among these key large-scale brain networks have been postulated as a model for cognitive impairment in psychotic psychopathology [17]. Nevertheless, the evidence, obtained largely from functional magnetic resonance imaging studies, is mixed and the specific cause of cognitive impairment in schizophrenia remains unclear [39]. It is also possible that psychosis and impaired cognitive operations are mediated by dual and separable but nonetheless highly comorbid pathologies. The generally weak or absent association between psychotic symptoms and cognitive performance [40] as well as the existence of a cognitively “normal,” or at least high-functioning, schizophrenia subpopulation suggests that dual process models are plausible [3].

It is also noteworthy that cortical thickness values associated with the central executive network did not differentiate patients and controls across or between levels of cognitive performance. This network tends to show increased activation during structured cognitive testing and associated cortical regions have long been implicated in the neural basis of schizophrenia [17]. Cortical thickness values may reflect several characteristics of intracortical morphology [41] and correlations between regions as demonstrated in our findings imply structural connectivity. However, these and similar data do not necessarily index physiological connectivity or activation patterns among networks in clinical populations [42]. Therefore, our results cannot be regarded as a definitive test of the importance of the central executive network in schizophrenia or in relation to cognitive impairment. In addition, regional overlap in our summed network thickness values means that these values were not independent. Prefrontal regions implicated in working memory and insular cortex involved in emotional-contextual processing were common to the central executive and salience networks [17]. The default mode and salience networks shared anterior cingulate subregions that contribute to executive function [18]. However, regional thicknesses were weighted equally and may not reflect their differential contribution to each network. These limitations make conclusions about the relative magnitude of cortical thinning in different networks in schizophrenia tentative and in need of further investigation.

In addition, the relatively small sample size of cognitively normal range patients may have reduced statistical power to detect significant differences relative to cognitively below-normal range patients in particular. However, the extremely small mean differences between patient groups (Cohen's* d*'s < 0.18) suggest that sample size alone was not the primary cause of nonsignificance in these comparisons. Therefore, cognitively high-functioning schizophrenia patients may indeed be indistinguishable from more typically impaired patients in terms of cortical thickness. Nonetheless, additional and alternate indices of neural structure and function should be considered in efforts to map this potentially informative behavioral distinction onto underlying brain.

## 5. Conclusions

Recent research suggests that cortical abnormalities including thinning, possibly reflecting reduced synaptic structure and excessive pruning during adolescence, are mediated by genes that increase the risk for developing schizophrenia [43]. This may help explain the progressive thinning reported in youth with elevated risk for psychosis [44]. It also implicates thinning as a neural feature of the illness shared across patients with differing clinical and cognitive profiles. Our data are consistent with this view and suggest that diffuse as well as more focal thinning in regions associated with the default mode and salience networks is specific to the psychotic disease process, whether or not it is accompanied by impairment in routine cognitive operations.

## Figures and Tables

**Table 1:** Descriptive and criterion data for cognitively normal range (CNR) and below-normal range (BNR) patients and controls.

Variable	CNR patients (*n* = 17)	CNR controls (*n* = 39)	BNR patients (*n* = 49)	BNR controls (*n* = 24)	Statistic
Age, years (M, SD)	34.47 (7.71)	37.46 (12.10)	43.90 (10.13)	41.17 (10.15)	*F*_3,125_ = 4.65^*∗∗*^
Education, years (M, SD)	14.53 (1.42)	13.51 (1.73)	12.35 (2.20)	10.79 (1.96)	*F*_3,125_ = 15.72^*∗∗∗*^
Gender (males %)	59	62	63	62	*χ*_3_^2^ = 0.11
MCCB composite *T* (M, SD)	46.94 (5.00)	50.51 (6.66)	23.31 (9.67)	26.54 (10.36)	*F*_3,125_ = 93.18^*∗∗∗*^
WRAT-4 Reading *SS* (M, SD)	100.53 (7.32)	101.18 (8.53)	87.87 (11.45)	84.83 (10.28)	*F*_3,123_ = 21.73^*∗∗∗*^
PANSS positive *T* (M, SD)	38.82 (6.19)	—	42.43 (7.90)	—	*t*_64_ = 1.70
PANSS negative *T* (M, SD)	37.29 (7.73)	—	39.24 (6.54)	—	*t*_64_ = 1.01
Medication (2nd generation)	76%		65%		*χ*_1_^2^ = 3.43

*Note*. MCCB: MATRICS Consensus Cognitive Battery; PANSS: Positive and Negative Syndrome Scale; WRAT-4: Wide Range Achievement Test.

^*∗∗*^ *p* < 0.01.

^*∗∗∗*^ *p* < 0.001.

**Table 2:** Cortical thickness (mm) in cognitively normal range (CNR) and below-normal range (BNR) patients and controls adjusted for age.

Region/network	(1) CNR patients (*n* = 17) M, SD	(2) CNR controls (*n* = 39) M, SD	(3) BNR patients (*n* = 49) M, SD	(4) BNR controls (*n* = 24) M, SD	*F*(3,123)	Bonferroni adjusted comparisons
Whole brain	2.46 (.10)	2.54 (.10)	2.46 (.10)	2.51 (.10)	5.56^*∗∗*^	2 > 1,3
Default mode	2.55 (.11)	2.68 (.11)	2.56 (.11)	2.62 (.11)	8.93^*∗∗∗*^	2 > 1,3
Salience	2.70 (.12)	2.81 (.12)	2.72 (.12)	2.77 (.12)	6.25^*∗∗*^	2 > 1,3
Central executive	2.55 (.13)	2.61 (.12)	2.57 (.13)	2.61 (.12)	1.65	

^*∗∗*^ *p* < 0.01.

^*∗∗∗*^ *p* < 0.001.
